# *Homatula guanheensis* sp. nov. (Teleostei: Nemacheilidae), a new species of loach from Henan Province, China

**DOI:** 10.3897/BDJ.9.e65130

**Published:** 2021-06-16

**Authors:** Chuanjiang Zhou, Wenwen Ma, Xi Wang, Yongtao Tang, Xiaoling Meng, Guoxing Nie

**Affiliations:** 1 Engineering Technology Research Center of Henan Province for Aquatic Animal Cultivation, College of Fisheries, Henan Normal University, Xinxiang City, China Engineering Technology Research Center of Henan Province for Aquatic Animal Cultivation, College of Fisheries, Henan Normal University Xinxiang City China

**Keywords:** *
Homatula
*, morphology, Hanjiang River drainage, taxonomy

## Abstract

**Background:**

The genus *Homatula* belongs to the order Cypriniformes and family Nemacheilidae. Nichols (1925) set up the genus as a subgenus of *Barbatula* by the type species of *Nemacheilus potanini*. Currently, it is recognised as a valid genus. Nineteen valid species have been already reported in the drainage of the Yellow, Yangtze, Pearl, Lancang, Red and Nujiang Rivers. *H. variegata*, *H. longidorsalis*, *H. berezowskii* and *H. potanini* are distributed in the Yangtze River drainage in China. *H. laxiclathra* is mainly distributed in the Weihe River, a tributary of the Yellow River. The remaining species are mainly distributed in the rivers of Yunnan Province.

**New information:**

*Homatula guanheensis* sp. nov., a new species, is described from the Guanhe River of the HanJiang River drainage (a tributary of the Yangtze River), Xixia County, Henan Province, China. It can be distinguished from its congeners by a combination of the following characters: the vertical brown bars on the body are wider than their interspaces, numbering 19–22; predorsal body partially scaled; the lateral line complete; adipose crest on caudal peduncle not reaching forward; the position of the anal-fin origin and the intestinal form. The new species displays distinct molecular divergence in the Cytochrome oxidase I (COI) and Cytochrome b (Cyt *b*) genes.

## Introduction

The Nemacheilid genus *Homatula* was established by Nichols in 1925 as a subgenus of *Barbatula*, based on the type species *Nemacheilus potanini*. Species from *Homatula* are small-size benthic fishes that are distributed in the drainage of the Yellow, Yangtze, Pearl, Lancang, Nujiang and Red Rivers. Some researchers have treated *Homatula* as a synonym of *Paracobitis* according to its adipose-crest (Chu and Chen 1990, Ding and Deng 1990, Min et al. 2010, Zhou and He 1993, Zhu 1989, Zhu and Cao 1988, Zhu and Wang 1985). However, the type species of *Paracobitis* was described from western Asia and these had a large geographical gap with the Chinese species (Bǎnǎrescu and Nalbant 1995, Kottelat 1990). Therefore, we accept the opinion of species in western Asia as *Paracobitis* and all species in China as *Homatula* (Kottelat 1990, Bǎnǎrescu and Nalbant 1995). Nine species of Homatula have been reported from China before 2010. *Homatula erhaiersis* (Zhu and Cao, 1988) was treated as a synonym of *H. anguillioides* (Zhu and Wang, 1985) in recent studies by the comparison of phylogenetic analysis and morphological characteristics (Min et al. 2012, Endruweit et al. 2018). In addition, an increasing number of new species of *Homatula* have been reported in recent years: *H. disparizona* (Min et al. 2013) was the first recorded species of *Homatula* from the Red River; *H. wenshanensis* (Yang et al. 2017) and *H. coccinocola* (Endruweit et al. 2018) were also found in the Red River; *H. change* (Endruweit 2015) was found in the upper Black River Basin; *H. wuliangensis* (Min et al. 2012) and *H. pycnolepis* (Hu and Zhang 2010) were collected from the Lancang River drainage; *H. nanpanjiangensis* (Min et al. 2010) was reported from the Pearl River; *H. laxiclathra* (Gu and Zhang 2011) was found in the Wei-He River of the Yellow River drainage in Shaanxi Province and three new species of *Homatula* were identified in the upper Salween River (Li et al. 2019).

The morphological variation of *Homatula* in the upper Yellow River drainage, the upper-middle reaches of Yangtze River drainage and the upper Pearl River drainage was investigated by Zeng et al. in 2012. However, the taxonomic status of the specimens from the Hanjiang River of the middle Yangtze River drainage were undetermined (Zeng et al. 2012). The specimens of *Homatula* from the Guanhe River (a tributary of the Han River) were collected during the investigation of fishery resources in Xixia County, Henan Province. Morphological data and DNA taxonomy (COI and Cyt *b*) were used to identify the samples and the analysis found differences with the species that had been previously discovered. An unnamed species, different from congeners, is described in this paper.

## Materials and methods

All specimens were examined and stored in the collection of the College of Fisheries, Henan Normal University, Xinxiang, Henan Province, China. For morphological analysis, 10 specimens were fixed in 10% formalin. All measurements and counts were made following Kottelat (1990). Measurements were made point to point with digital calipers to the nearest 0.1 mm. X-ray films were used to count simple fin rays. Comparative morphometry was examined using PCA in IBM SPSS Statistics 22.0. The PCA analysis was processed using log-transformed morphometric data of percent of Standard Length (SL) in the variance-covariance matrix. To compare molecular characters, eighteen specimens were fixed in 95% ethanol and total genomic DNA was extracted from muscle tissue using standard phenol-chloroform extraction protocols (Sambrook et al. 1989). Two pairs of primers were used to amplify segments of COI and Cyt *b* by the polymerase chain reaction. The COI primers were designed with reference to other Osteichthyes COI gene sequences: Fish-CO I-F (5’-TCT CAA CCA ACC ATA AAG ACA TTGG-3’); Fish-CO I-R (5’-TAT ACT TCT GGG TGC CCA AAG AAT CA-3’). The Cyt *b* gene primers were designed as GluF (5’-AAC CAC CGT TGT ATT CAA CTA CAA-3’); ThrR (5’-ACC TCC GAT CTT CGG ATT ACA AGA CCG-3’) (Machordom and Doadrio 2001). PCR reactions were carried out in a 30 μl volume and amplifications proceeded for 5 min at 94°C followed by 34 cycles of 95°C for 30 s, 55°C for 30 s, 72°C for 60 s and final extension at 72°C for 10 min. The PCR products were sent to a commercial corporation for sequencing. The nucleotide sequences were assembled from independent sequence passes using the SeqMan (Swindell and Plasterer 1997) module of the DNAStar software package. Additionally, sequences for other species of *Homatula* were obtained from GenBank (Endruweit et al. 2018, Min et al. 2012, Xiong et al. 2017, Yue et al. 2013). Thirteen species of *Homatula* were studied, from which 680-bp COI sequences of 50 samples and 1,120-bp Cyt *b* sequences of 42 samples were used to identify the new species. A list of taxa with corresponding GenBank accession numbers is presented in Table 1. Phylogenetic analysis was performed using PhyloSuite (Zhang et al. 2020). Multiple alignments were performed with MAFFT version 7 with the default parameters (Katoh and Standley 2013). Then, ModelFinder (Kalyaanamoorthy et al. 2017) was used to ascertain the best-ﬁt model of nucleotide substitution for the sequences using the AIC. Tree analyses were performed under Bayesian Inference (BI). All Bayesian phylogenetic analyses were conducted with MrBayes 3.2 (Ronquist et al. 2012), based on the GTR+F+G4 model estimated by ModelFinder. Four Markov chains were run for 1,000,000 generations to estimate the posterior probability distribution, sampling every 1000 generations. After discarding the ﬁrst 1000 trees as a burn-in with non-stationary log likelihood values, 50% majority-rule consensus trees were estimated for the remaining trees. Finally, all sequences were grouped according to the results of the phylogenetic analysis. The genetic distances between groups were calculated using MEGA7.0 (Kumar et al. 2016), based on the K2P model.

## Data resources

All the sequences in this study were retrieved from GenBank and the accession numbers of the newly determined sequences in this study are MT771689-MT771705 (COI) and MT771706-MT1722 (Cyt *b*).

## Taxon treatments

### Homatula guanheensis

C. J. Zhou, W. W. Ma, Xi Wang, Y.T. Tang, X.L. Meng and G.X. Nie, 2021 sp. n.

48D0735E-DCEA-58E1-BFFD-C6879CB85666

5B17C202-F739-4EB6-BF82-1FF6CDCA25A9

#### Materials

- **Type status:** Holotype. **Occurrence:** recordNumber: HNU 010048; recordedBy: Henan Provincial Fish Resources Investigation Team; individualCount: 1; sex: female; lifeStage: adult; behavior: cave environment scoured by flowing water; **Taxon:** scientificName: *Homatula guanheensis*; kingdom: Animalia; phylum: Chordata; class: Actinopterygii; order: Cypriniformes; family: Nemacheilidae; subgenus: Homatula; **Location:** waterBody: the Yangtze River; country: China; stateProvince: Henan Province; county: Xixia County; locality: the Guanhe River, a tributary of the Hanjiang River drainage; verbatimElevation: 521 m a.s.l.; verbatimCoordinates: 33°52′79.3″N 111°70'94''E; georeferenceSources: Google Earth; **Identification:** identifiedBy: Chuan-Jiang Zhou; dateIdentified: 03/21/2017; **Event:** eventDate: 21/03/2017; **Record Level:** collectionCode: fish; basisOfRecord: Preserved Specimen
- **Type status:** Paratype. **Occurrence:** recordNumber: HNU 010049-HNU 010056; recordedBy: Henan Provincial Fish Resources Investigation Team; individualCount: 8; lifeStage: adult; behavior: cave environment scoured by flowing water; **Taxon:** scientificName: *Homatula guanheensis*; **Location:** waterBody: the Yangtze River; country: China; stateProvince: Henan Province; county: Xixia County; locality: the Guanhe River, a tributary of the Hanjiang River drainage; verbatimElevation: 522 m; verbatimCoordinates: 33°52′79.3″N 111°70'95''E; georeferenceSources: Google Earth; **Identification:** identifiedBy: Chuan-Jiang Zhou; dateIdentified: 03/22/2017; **Event:** eventDate: 21/03/2017; **Record Level:** collectionCode: fish; basisOfRecord: Preserved Specimen
- **Type status:** Paratype. **Occurrence:** recordNumber: HNU 010060; recordedBy: Henan Provincial Fish Resources Investigation Team; individualCount: 1; sex: female; lifeStage: adult; behavior: cave environment scoured by flowing water; **Taxon:** scientificName: *Homatula guanheensis*; **Location:** waterBody: the Yangtze River; country: China; stateProvince: Henan Province; county: Xixia County; locality: the Guanhe River，a tributary of the Hanjiang River drainage; verbatimElevation: 523 m; verbatimCoordinates: 33°52′79.3″N 111°70'96''E; georeferenceSources: Google Earth; **Identification:** identifiedBy: Chuan-Jiang Zhou; dateIdentified: 23/03/2017; **Record Level:** collectionCode: fish; basisOfRecord: Preserved Specimen

#### Description

**Figure 1.**
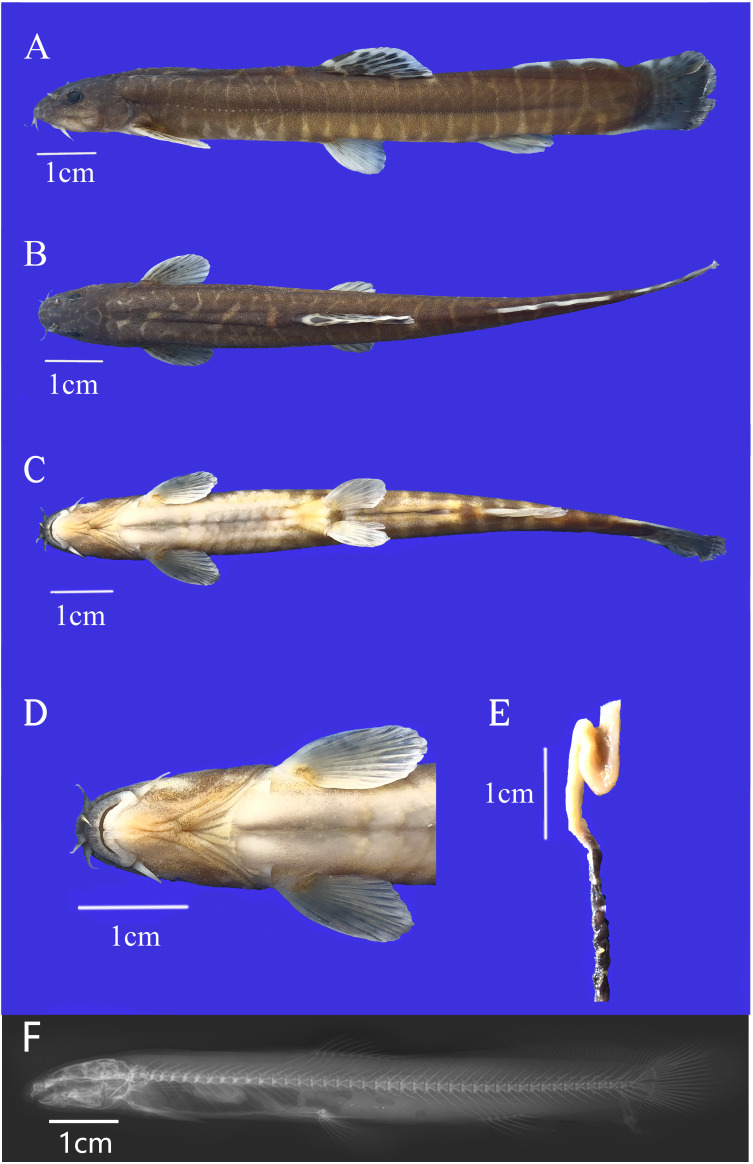
*Homatula guanheensis* (holotype, HNU 010048, 99.6 mm SL). **A.** Lateral view; **B.** Dorsal view; **C.** Ventral view; **D.** Mouth characters; **E.** Intestine form; **F.** X–ray (lateral view).

Body elongate, anterior portion nearly cylindrical and posterior portion compressed; body depth 11.63% (10.41–13.47%) in SL (Table 2). Body scales, back and sides of post-dorsal body closely covered by small scales, predorsal body scales sparse; head, thorax and abdomen scaleless. Lateral line straight, complete and mid-lateral. Vertebrae count 4+41–43. Head short and depressed, naked, wider 56.07% (49.39%–61.12%) in HL than high 45.77% (42.42%–49.95%) in HL. Snout blunt, length 40.75% (34.48%–44.35%) in HL (Table 2). Anterior nostril forms a valve, nostril closer to anterior margin of eye than to snout tip. Eyes oval, closer to snout tip, indiscernible from ventral view. Interorbital width 28.47% (23.89%–31.09%) in HL (Table 2). Mouth inferior, lips thick and furrowed, jaws covered by lips, upper jaw with developed processus dentiformis corresponding with marked median notch on lower jaw. Three pairs of barbels: two rostral barbels, inner pair not reaching mouth corner and outer pair reaching vertical line of anterior nostril; one maxillary barbel extending to the middle and posterior margin of eye (Fig. 1). Dorsal fin ⅲ, 7–8^1/^_2_ rays, origin nearer to snout tip than to caudal-fin base. Pectoral fin ⅰ, 9–10 rays, not extending beyond halfway from its origin to the pelvic-fin origin. Pelvic fin ⅰ, 6–7 rays, its origins closer to vertical line of first branched rays of dorsal fin; tip of the pelvic fin not extending beyond half the distance from its origin to anal fin origin. Anal fin ⅲ, 5^1/^_2_ rays; origin of anal fin closer to pelvic fin origin than to caudal fin base, its tip not reaching half distance from anal-fin origin to caudal-fin base. Posterior margin of caudal fin micro-rounded; adipose crests along its dorsal and ventral mid-lines without extending through the origin of anal fin. Intestine formed as a bend, not reaching posterior surface of the U-shaped stomach. Gas bladder osseous, anterior chamber invisible, fully enclosed in a capsule; posterior chamber degenerated.

##### Color in preserved specimens (fixed in 10% formalin)

Head and body brown; a series of 19–22 body bars, each bar at least twice as wide as the interspace. Abdomen yellowish. Dorsal fin with two dark brown marks, one at the base, the other at postmedian of the fin; posterior border of dorsal fin white. Pectoral fins with dark brown spots. Pelvic and anal fins white, dark at the base. Adipose keels white with dark brown spots. Caudal fin dark grey; brown vertical bars on caudal fin base (Fig. 1).

#### Diagnosis

*Homatula guanheensis* is different from its congeners in the following characters: partly scaled (vs. in the latter, scales are totally absent or only a few scales on the caudal peduncle in *H. nanpanjiangensis*, *H. oligolepis*, *H. disparizona* and *H. wenshanensis* vs. scales all over the body, except for the head in *H. acuticephala*, *H. anguillioides*, *H. pycnolepis*, *H. wuliangensis*, *H. change* and *H. coccinocola*); complete lateral line (vs. incomplete lateral line in *H. potanini* and *H. wujiangensis*); the vertical brown bars on the body are wider than their interspaces, numbering 19–22 (vs. equal to interspace or slightly wider than its interspace in *H. variegate*, *H. berezowskii* and *H. longidorsalis*); caudal fin micro-rounded (vs. truncated or oblique in *H. variegata* and *H. berezowskii*); adipose crest on caudal peduncle not reaching forward of the position of the anal-fin origin (vs. beyond in *H. variegate* vs. identical in *H. longidorsalis*); predorsal body partially scaled (vs. absent scales in *H. berezowskii* and *H. longidorsalis*); anterior nostril forming a valve (vs. forming a spool in *H. longidorsalis*). The vertical brown bars on the body of the new species are similar to *H. laxiclathra*. The new species can be further distinguished from *H. laxiclathra* in that the intestine forms a bend, not reaching the posterior surface of U-shaped stomach (vs. a loop anteriorly reaching the posterior surface of the U-shaped stomach); anterior with only a few and scattered scales (vs. scaleless).

#### Etymology

The specific epithet *Guanheensis* is derived from Guanhe River (鹳河 in Chinese, type locality) with the Latin suffix "-ensis".

#### Distribution

*Homatula guanheensis* sp. nov. is known from the Guanhe River of the Hanjiang River drainage (a tributary of the Yangtze River) in Henan Province, Central China (Fig. 3).

## Identification Keys

### Keys to species of the genus *Homatula* in China

**Table:** 

1	Lateral line incomplete	2
–	Lateral line complete	3
2	Scales present; covering whole body except head and abdomen; dorsal crest high and long, from dorsal-fin base to caudal-fin base (Jinshajiang River, Sichuan Province)	*H. potanini* (Günther, 1896)
–	Body scaleless or with rudimentary scales; dorsal crest high and short, upper crest not reaching the posterior point of anal-fin base (Wujiang River, Sichuan Province)	*H. wujiangensis* (Ding & Deng, 1990)
3	Anterior ending of adipose crest does not reach posterior end of anal-fin base	4
–	Anterior ending of adipose crest reach posterior end of anal-fin base	7
4	The length of dorsal-fin base longer than the longest branched dorsal-fin ray (Nujiang River, Yunnan Province)	*H. cryptoclathrata* (Li et al., 2019)
–	The length of dorsal-fin base shorter than the longest branched dorsal-fin ray	5
5	Having clear pattern of marks on the flank	6
–	No marks on the flank (Nujiang River, Yunnan Province)	*H. nigra* (Li et al., 2019)
6	27-34 marks on the flank; dorsal and pelvic fins closer to snout (Nujiang River, Yunnan Province)	*H. anteridorsalis* (Li et al., 2019)
–	20-22 marks on the flank; dorsal and pelvic fins located intermediate of body (Mengyejiang River, Yunnan Province)	*H. change* (Endruweit, 2015)
7	Scales are totally absent or only a few scales on the caudal peduncle	8
–	Scales clearly present, covering posterior of body at least	11
8	No median notch on lower jaw	9
–	Median notch on lower jaw	10
9	The caudal fin emarginate; caudal peduncle narrow and long; vertebrae 4+39~40 (Panlong River, Yunnan Province)	*H. disparizona* (Min et al., 2013)
–	The caudal fin furcate; caudal peduncle wider and shorter; vertebrae 4+47~48 (the Red River, Yunnan Province)	*H. wenshanensis* (Yang et al., 2017)
10	Body with regular vertical bars and bars in front of dorsal fin conspicuously thinner than those behind; no vermiform markings on parietal area or obscure; tip of pelvic fin closing or reaching anus (Nanpanjiang River, Yunnan Province)	*H. nanpanjiangensis* (Min et al., 2010)
–	Body and head with vermiform markings; dorsal fin and pectoral fin covered by small spots on both sides; tip of pelvic fin quite far away from anus (Yangzonghai River, Yunnan Province)	*H. oligolepis* (Cao & Zhu, 1989)
11	Scales covering whole body, except head	12
–	Posterior part of the body covered by scales; anterior part scaleless or with only a few and scattered scales	16
12	Having pelvic axillary lobe	13
–	Absent pelvic axillary lobeAbsent pelvic axillary lobe	15
13	A pair of free protrusions present in pelvic fins (Yangbijiang River, Yunnan Province)	*H. pycnolepis* (Hu & Zhang, 2010)
–	Absent free protrusions in pelvic fins	14
14	Caudal fin slightly emarginated to nearly truncate; having notch on the lower jaw; 16-19 brown bars on a beige background, bars are somewhat straight and never vertically split, notch on the lower jaw (Tengtiaojiang River, Yunnan Province)	*H. coccinocola* (Min et al., 2018)
–	Caudal fin rounded; lacking notch on the lower jaw; 22-26 brown bars on body (Lancang River, Yunnan Province)	*H. wuliangensis* (Min et al., 2012)
15	Caudal fin truncated; body depth extremely decreased posterior of dorsal-fin base; head sharp (Haixihai Lake, Yunnan Province)	*H. acuticephala* (Zhou & He, 1993)
–	Caudal fin oblique; body depth quite uniform from head to tail; head blunt (Mekong River, Yunnan Province)	*H. anguillioides* (Zhu & Wang, 1985)
16	Anterior nostrils pierced in front side of a tube; dorsal fin is far from the snout (Nanpangjiang River, Yunnan Province)	*H. longidorsalis* (Yang et al., 1989)
–	Anterior nostrils pierced in front side of a flap, dorsal fin closer to the snout	17
17	Vertical brown bars narrower or slightly wider than their interspaces	18
–	Vertical brown bars distinctly wider than interspaces	19
18	Caudal fin oblique; intestine forming a single loop; adipose crest of the caudal peduncle anteriorly extending through the anal-fin origin (Jinshajiang River, Weihe River)	*H. variegata* (Sanvage & Dabry, 1874)
–	Caudal fin truncate; intestine forming a zigzag loop; adipose crest of the caudal peduncle anteriorly not extending through the anal-fin origin (Jialingjiang River, Gansu Province)	*H. berezowskii* (Günther, 1896)
19	Caudal fin oblique; intestine with a loop anteriorly reaching the posterior surface of the U-shaped stomach; anterior scaleless (Weihe River, Shaanxi Province)	*H. laxiclathra* (Gu & Zhang, 2012)
–	Caudal fin micro-rounded; intestine forming a bend; anterior with only a few and scattered scales (Guan River, Henan Province)	*H. guanheensis* sp. nov.

## Analysis

**Genetic distance and phylogentic trees**

Based on the COI gene, sequences of 11 studied species were used to construct a BI tree. The new species belonged to a different clade with strong high PP values (100%, Fig. 4), the genetic distance being analysed by the Kimura 2-parameter model. The distance matrix and number of sequences, determined for each species, are shown in Table 4 and Table 5. Genetic distance between *H. guanheensis* and other given species displayed 4.7%–10.6% variation in the COI base pairs (Table 4). The smallest distance, 2.9% (5 vs. 9) was detected between *H. pycnolepis* and *H. anguillioides*. Based on the Cyt *b* gene, the phylogenetic tree analysis showed that *H. guanheensis* is a monophyletic group with a high posterior probability value (100%; Fig. 5). Through the results of the genetic distance analysis, we found that *H. guanheensis* differed from the acquired congeners by 4.2%–10.3%. The genetic distance between *H. guanheensis* and similar species exceeded 2% variation, while the intraspecific variation of *H. guanheensis* was 0% (Table 5).

## Discussion

**Figure 2.**
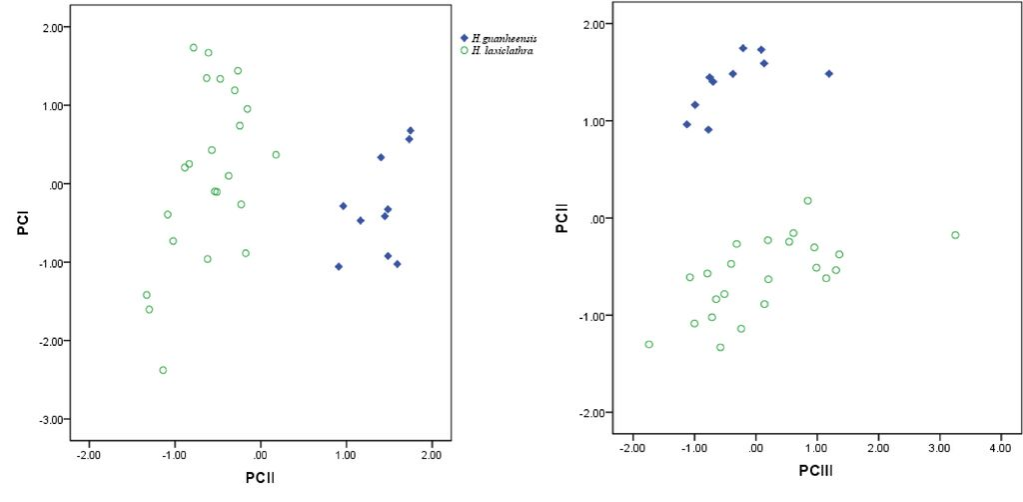
Scatter plots of the first and second principal components from morphometric data of *H. guanheensis* (n = 10, 76.9 –109.26 mm SL) and *H. laxiclathra* (n = 23, 67.6–136.7 mm SL).

**Figure 3.**
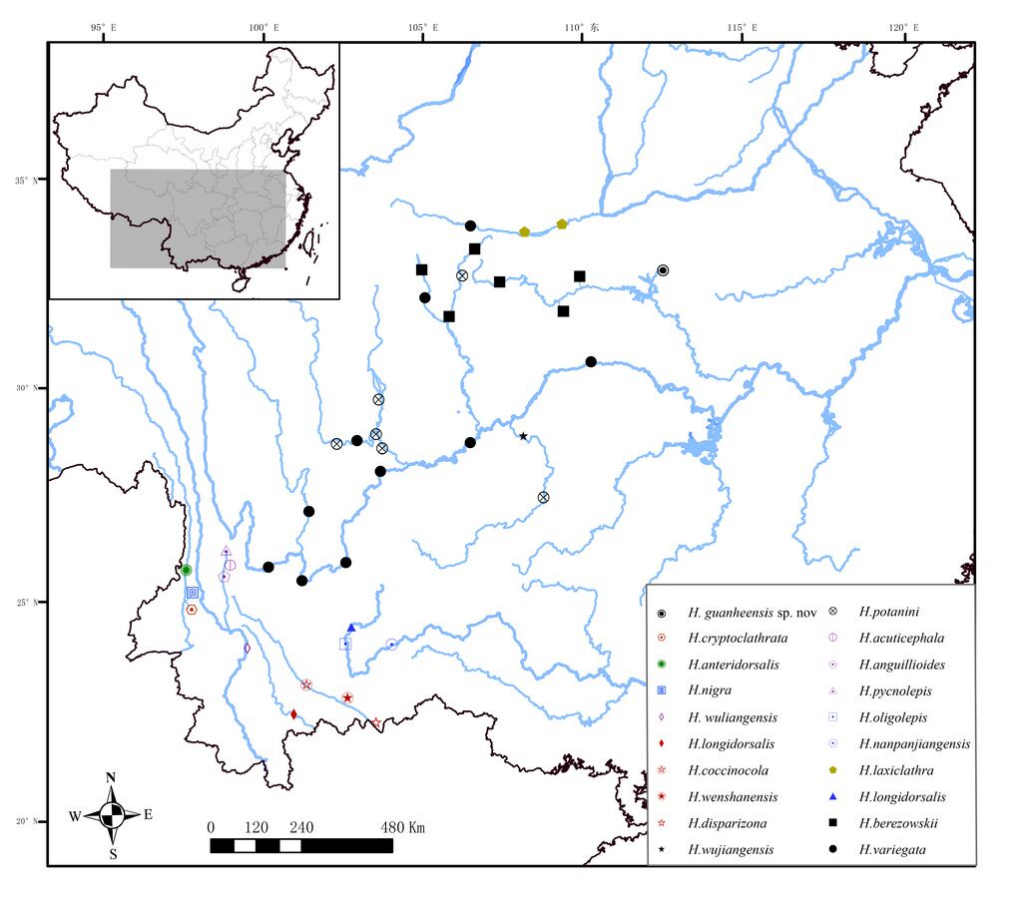
Distribution of all species of *Homatula* in China.

New species were collected from the Guanhe River of the Hanjiang River drainage (the middle and lower reaches of the Yangtze River). In terms of geographic distribution, the known species in the Yangtze River Basin are as follows: *Homatula variegata*, *H. wujiangensis*, *H. berezowskii* and *H. potanini*. *H. variegata* and *H. potanini* are mainly distributed in the upper reaches of the Yangtze River, while *H. berezowskii* is mainly distributed in the Jialingjiang River and the Hanjiang River (Zhu 1989, Yang et al. 1994, Hu and Zhang 2010). Zeng studied the samples of *Homatula* in the Yangtze River and the Yellow River and pointed out that there were differences in interorbital width and caudal peduncle length between *H. berezowskii* specimens of the Hanjiang River and the Jialingjiang River (Zeng et al. 2012). The new species was initially thought to belong to *H. berezowskii*; however, this hypothesis was quickly abandoned due to the differences in morphology and molecular characters. *H. guanheensis* is distinguished from *H. berezowskii* in having bars at least twice as wide as the interspace (vs. equal to the interspace or slightly wider than its interspace); scales partly on anterior body (vs. scaleless); intestine flat (vs. zigzag); a higher caudal peduncle, depth 11.3% (10.1%–13.6%) in SL [vs. 10.0% (9.1%–10.9%)] (Suppl. material 1). Besides this, they have significant divergence in genetic distance (4.2%, Cyt *b*). The new species can be distinguished from other species in the Yangtze River Basin by the characteristics of the vertical brown bars, body scale distribution, adipose crest, intestine form and dorsal fin positions. *H. guanheensis* can be clearly distinguished from *H. potanini* and *H. wujiangensis* from the lateral line (complete vs. incomplete). It is distinct from *H. variegata* by having wider bars (vs. narrower), adipose keels not beyond the origin of anal-fin (vs. beyond), head flat, depth 45.8% (42.2%–49.9%) in HL [vs. 49.4% (43.7%–56.8%)] (Suppl. material 1). Besides, the vertical brown bars on the body of the new species are similar to *H. laxiclathra*. The first two Principal Components of a processed Principal Component Analysis explained 85% of the variance (Fig. 2Table 3). Meanwhile, the new species can be further distinguished from *H. laxiclathra* in having a flat intestine, not reaching posterior surface of the U-shaped stomach (vs. a loop anteriorly and reaching posterior surface); scales partly on anterior body (vs. scaleless); a wider interorbital width 28.5% (23.9%–31.1%) in HL [vs. 22.5% (19.9%–24.9%)]; a higher caudal peduncle, depth 11.3% (10.1%–13.6%) in SL [vs. 9.2% (8.4%–11.4%)] (Suppl. material 1). Similar to *H. longidorsalis*, the new species has a slender body with uniform depth, while it differs from *H. longidorsalis* by having scales on the predorsal body (vs. scaleless on the predorsal body); anterior nostril forming a valve (vs. forming a spool); vertical brown bars at least twice as wide as interspace (vs. bars wider than interspace on anterior portion, unclear on posterior portion); adipose keels not beyond the origin of anal-fin (vs. identical); a shorter head, length 18.7% (17.3%–20.3%) in SL [vs. 23.2% (21.5%–25.3%)]; pelvic-fin close to tip of snout, prepelvic length 46.7% (43.9%–49.33%) in SL [vs. 52.6% (50.5%–56.7%)] (Suppl. material 1).

**Figure 4.**
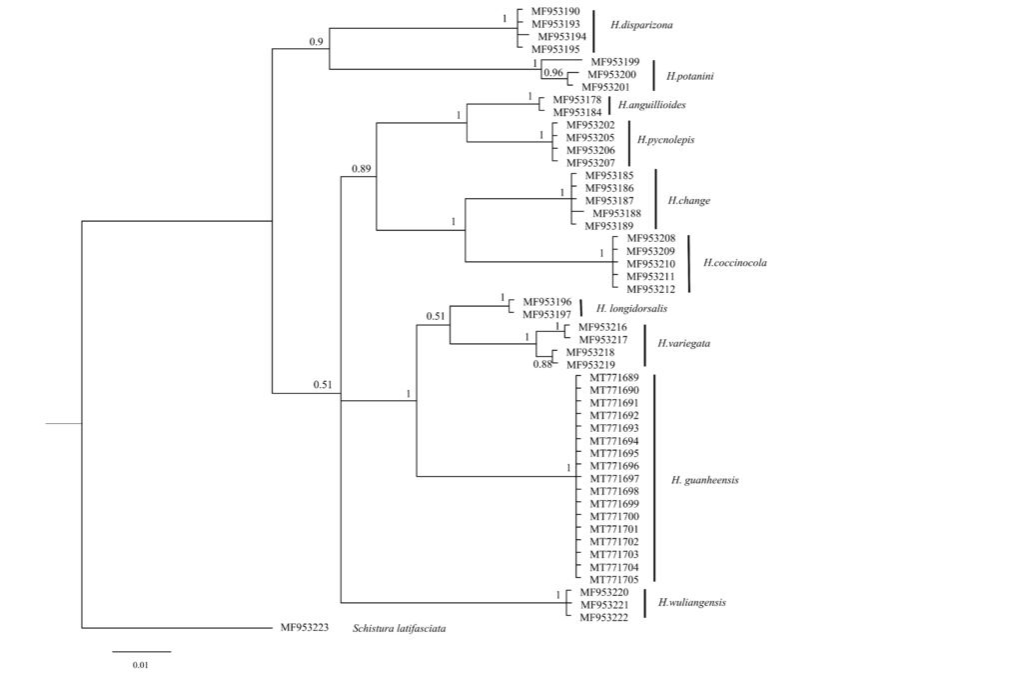
The phylogenetic tree of *Homatula* based on the COI gene; *Schistura latifasciata* was used as the outgroup.

**Figure 5.**
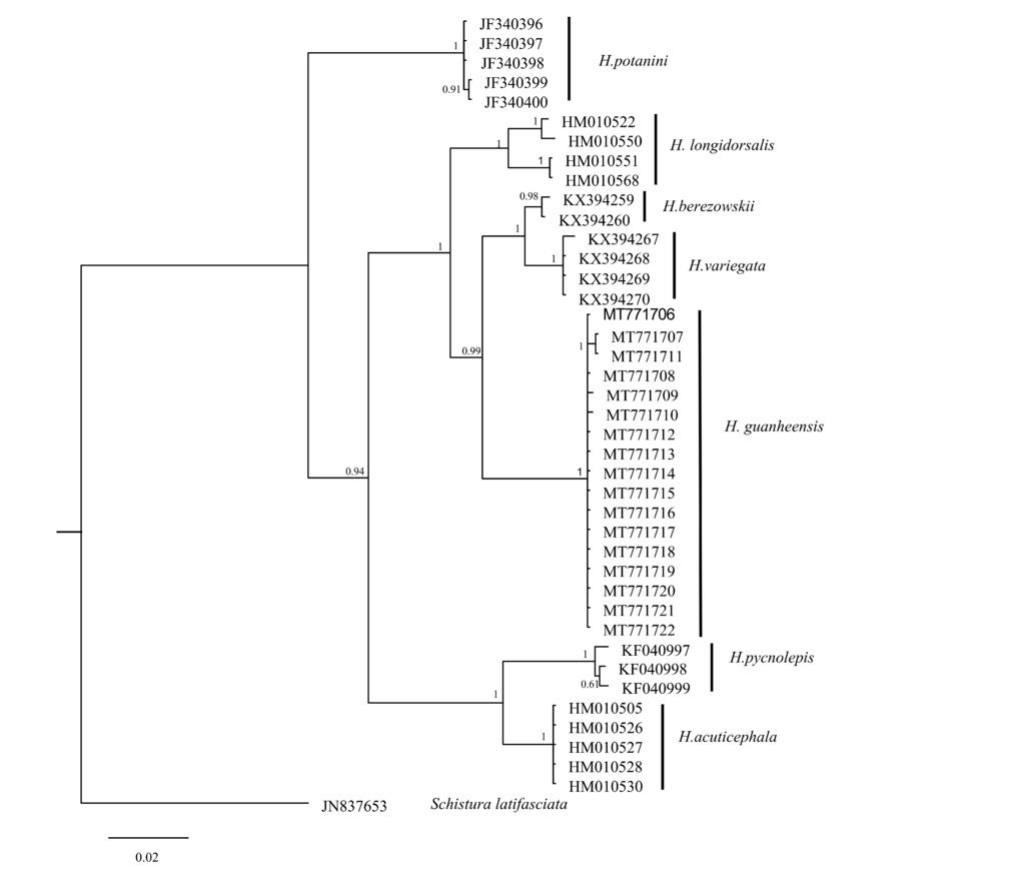
The phylogenetic tree of *Homatula* based on the Cyt *b* gene; *Schistura latifasciata* was used as the outgroup.

According to the results of the phylogenetic tree, *H. variegata*, *H. berezowskii*, *H. longidorsalis* and the new species gather into a branch, *H. longidorsalis* and *H. guanheensis* + (*H. berezowskii* + *H. variegata*) form a sister group (supported by the Cyt *b* gene, Fig. 5), but the new species and *H. longidorsalis* + *H. variegata* form a sister group (supported by the COI gene, Fig. 4). The cause might vary amongst datasets and, thus, this discordance might be influenced by stochastic errors associated with a different number of species and datasets (Naylor and Brown 1998). The results of Cyt *b* data and the COI data show that the new species has a far genetic distance from *H. berezowskii*, *H. variegata* and *H. longidorsalis*.

## Supplementary Material

27A530CC-9099-5962-BB56-6C8BFC5C4AE9 10.3897/BDJ.9.e65130.suppl1 Supplementary material 1 Morphometric characters of *Homatula variegata*, *H. berezowskii*, *H. longidorsalis* and *H. laxiclathra* from Gu and Zhang (2011). Data type morphological Brief description Morphometric characters of *Homatula variegata*, *H. berezowskii*, *H. longidorsalis* and *H. laxiclathra* from Gu and Zhang (2011), comparative materials with Table 2. File: oo_513071.xlsx https://binary.pensoft.net/file/513071 Gu and Zhang

XML Treatment for Homatula guanheensis

## Figures and Tables

**Table 1.** GenBank accession numbers of COI and Cyt *b* sequences.

**Species**	**COI Gene Accession Numbers**	**Cyt *b* Gene Accession Numbers**
*H. longidorsalis*	MF953196*	HM010550*
	MF953197*	HM010551*
		HM010552*
		HM010568*
*H. anguillioides*	MF953178*	
	MF953184*	
*H. acuticephala*		HM010526*
		HM010505*
		HM010527*
		HM010528*
*H. berezowskii*		KX394259*
		KX394260*
*H. coccinocola*	MF953208*	
	MF953209*	
	MF953210*	
	MF953211*	
	MF953212*	
*H. disparizona*	MF953190*	
	MF953193*	
	MF953194*	
	MF953195*	
*H. potanini*	MF953199*	JF340396*
	MF953200*	JF340397*
	MF953201*	JF340398*
		JF340399*
		JF340400*
*H. pycnolepis*	MF953202*	KF040997*
	MF953205*	KF040998*
	MF953206*	KF040999*
	MF953207*	
*H. variegata*	MF953216*	KX394267*
	MF953217*	KX394268*
	MF953218*	KX394269*
	MF953219*	KX394270*
*H. wuliangensis*	MF953220*	
	MF953221*	
	MF953222*	
*H. guanheensis* sp. nov	MT771689-MT771705	MT771706-MT1722
*Schistura latifasciata*	MF953223*	JN837653*

* Sequences were retrieved from GenBank.

**Table 2.** Trials and morphometric characters of *Homatula guanheensis* sp. nov.

Holotype			Paratypes (n = 9)		
			range	mean	SD
Standard length (mm)	99.6			76.9-109.26	91.8	
**As%SL**						
Body depth	11.6			10.4-13.4	11.9	0.8
Head length	18.5			17.3-20.3	18.7	0.9
Dorsal-fin length	**18.5**			**17.3-21.3**	**19.3**	**1.3**
Pectoral-fin length	12.9			11.4-14.2	12.7	0.9
Pelvic-fin length	10.5			10.3-12.7	11.4	0.8
Anal-fin length	**13.4**			**11.6-14.1**	**13**	**0.8**
Predorsal length	46.5			42.3-49.1	45.7	2.3
Pre-anus length	18.1			16.4-20.2	18.4	1.3
Prepelvic length	47.2			43.9-49.3	46.7	1.2
Pre-anal length	72.7			68.8-73.4	71.1	2
Caudal peduncle depth	13.6			10.1-13.6	11.3	1.2
Caudal peduncle length	20.9			16.4-23.6	20.2	1.8
**As%Caudal peduncle length**						
Caudal peduncle depth		**65.1**	**45.7-76.8**		**56.6**	**8.9**
**As%HL**						
Head depth		43.4	**42.4-49.9**		**45.8**	**2.6**
Head width		53.7	49.3-61.1		56.1	3.7
Eye diameter		14.6	12.2-16.9		14.6	1.2
Snout length		**40.1**	**34.4-44.3**		**40.7**	**3.5**
Interorbital width		**29.9**	**23.8-31.1**		**28.5**	**2.2**

**Table 3.** Component matrix of the Principal Component Analysis from morphometric data of *H. guanheensis* and *H. laxiclathra*.

	PC1	PC2	PC3
Body depth	0.156	0.126	0.889
Head length	0.851	0.264	0.229
Head depth	0.515	-0.088	0.46
Head width	0.136	-0.074	0.099
Eye diameter	0.807	-0.041	0.384
Interorbital width	0.137	0.91	0.045
Snout length	0.701	0.148	0.036
Dorsal-fin length	0.015	0.976	-0.172
Pectoral-fin length	0.78	0.094	0.31
Pelvic-fin length	0.683	0.527	0.196
Anal-fin length	0.182	0.961	-0.078
Predorsal length	0.346	0.054	0.724
Prepelvic length	0.292	-0.09	0.753
Prepectoral length	0.481	0.095	0.606
Pre-anal length	0.621	-0.161	0.333
Caudal peduncle length	-0.235	0.817	0.229
Caudal peduncle depth	-0.681	0.111	-0.447
Cumulative variance (%)	59.35	79.65	85.10

**Table 4.** Pairwise comparisons of genetic distance of nine *Homatula* species, based on K2P model from sequences of the COI gene, with number of sequences per species (n), the intraspecific variation, followed by the distance between species (in %).

Species	n	intraspecific variation	2	3	4	5	6	7	8	9	10	11
*H. guanheensis*	17	0.0	8.2	7.7	5.1	7.9	8.7	4.7	8.1	8.3	7.6	10.6
*H. coccinocola* (2)	5	0.0		8.0	7.1	7.0	9.7	6.5	8.9	6.9	4.9	11.9
*H. wuliangensis* (3)	3	0.0			6.5	7.3	9.8	6.7	8.9	7.5	6.3	10.5
*H. variegata* (4)	4	0.5				7.2	8.1	3.2	7.4	6.8	6.2	10.5
*H. pycnolepis* (5)	4	0.0					7.2	6.2	8.4	2.9	6.4	10.0
*H. potanini* (6)	3	0.9						7.2	8.0	7.4	8.4	10.2
*H. longidorsalis* (7)	2	0.0							7.1	6.2	5.9	9.6
*H. disparizona* (8)	4	0.1								8.3	8.3	10.0
*H. anguillioides* (9)	2	0.0									6.2	10.3
*H. change* (10)	5	0.0										11.1
*S. latifasciata* (11)	1											

**Table 5.** Pairwise comparisons of genetic distance of six *Homatula* species, based on K2P model from sequences of the Cyt *b* gene, the other annotations being the same as Table 4.

Species	n	intraspecific variation							
			12	13	14	15	16	17	18
*H. guanheensis*	17	0.1	4.8	10.3	9.9	4.2	5.8	9.5	15.8
*H. variegata* (12)	4	0.1		9.8	9.3	1.6	4.9	9.3	14.7
*H. pycnolepis* (13)	3	0.4			10.3	9.5	9.2	4.0	15.4
*H. potanini* (14)	5	0.1				8.7	8.7	9.2	13.6
*H. berezowskii* (15)	2	0.2					4.7	8.5	14.5
*H. longidorsalis* (16)	4	1.5						8.2	14.3
*H. acuticephala* (17)	5	0.0							14.3
*S. latifasciata* (18)	1								
